# Supplementary material for: Safety of Fingolimod in Patients with Multiple Sclerosis Switched from Natalizumab: Results from TRANSITION―A 2-Year, Multicenter, Observational, Cohort Study
Source: Brain Sci. 2022 Feb 4;12(2):215. doi: 10.3390/brainsci12020215 (PMC8870332; doi:10.3390/brainsci12020215)
Supplement: Supplementary file 1 [file brainsci-12-00215-s001.zip › brainsci-1508471-SI.pdf]

Supplementary material

Methods

Annualized relapse rate (ARR) is defined as the number of relapses with onset occurring during a specific period of time, adjusted to a one-year period. The group-level ARR was calculated by total number of relapses experienced by all patients in the group during a specified time period, divided by the total number of days in that specified time period, and the ratio multiplied by 365.25. Patient-level ARR was calculated by the number of relapses experienced by that patient divided by the number of days the patient participated in the study, and the ratio multiplied by 365.25.

Results from subgroup analysis

Overall, 67.3% of patients from the prospective and 69.9% from the retrospective group switched to fingolimod from natalizumab owing to their JCV seropositivity. Similarly, 27.4% in the prospective group and 34.6% in the retrospective group switched to fingolimod because of >2 years of treatment duration with natalizumab.

A total of 188 relapses were observed in 117 prospective patients and 144 relapses were observed in 85 retrospective patients. Majority of relapses were treated in both groups (prospective group, 167 [88.8%]; retrospective group, 120 [83.3]) and 73.9% and 78.5% of patients from prospective and retrospective groups, respectively, recovered either completely or partially.

Figure S1. TRANSITION study design.

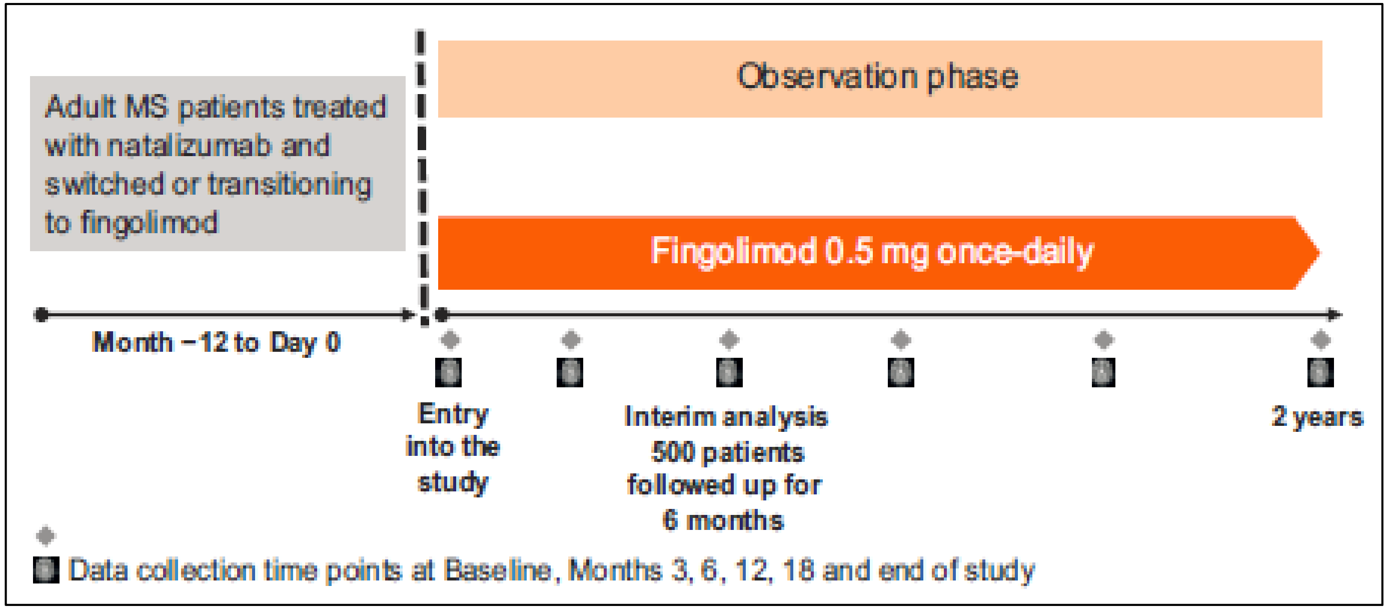

Table S1. Patient enrollment centers.

Center Country	No. of Pts Enrolled	No. of sites
Germany	85	35
United States	233	50
Canada	28	4
Greece	19	5
Israel	12	2
Italy	133	15
Portugal	2	1
United Kingdom	31	7
Ireland	16	3
Belgium	59	9
Norway	24	5

Table S2. Occurrence of AEs and SAEs.

Subgroup		AEs		SAEs	
		**ORR (95% CI)**	**P value**	**ORR (95% CI)**	**P value**
**Natalizumab washout period**	**≤8 weeks**<br>**>8 weeks**	1.093 (0.848, 1.409)	0.4915	0.910 (0.467, 1.774)	0.7828
Natalizumab exposure	**≤2 years**<br>**>2 years**	0.974 (0.779, 1.220)	0.8215	0.883 (0.493, 1.582)	0.6757
Exposure to prior immunosuppressive agents	**No**<br>**Yes**	1.848 (1.017, 3.358)	0.0440	0.952 (0.224, 4.038)	0.9469
JCV status	**Negative**<br>**Positive**	1.327 (0.988, 1.782)	0.0602	1.735 (0.835, 3.606)	0.1400

Table S3. Frequent AEs and SAEs reported in different subgroups (safety set).

**Subgroup**		AEs, n (%); IR	Commonly reported AEs		SAEs, n (%); IR	Commonly reported SAEs
			**AEs >5%**	**n (%); IR**		**n; IR**
**Natalizumab washout period**	**≤8 weeks (N=156)**	118 (75.6); 107.8	Fatigue	25 (16.0; 9.5	14 (9.0); 4.9	>1 patient<br><br>UTI (n=2; 0.67)<br>MS relapse (n=2; 0.67)
			Diarrhea	16 (10.3); 5.7		
			Lymphopenia	13 (8.3); 4.5		
			Headache	13 (8.3); 4.6		
			UTI	12 (7.7), 4.2		
			Paresthesia	10 (6.4); 3.5		
			Nasopharyngitis	10 (6.4); 3.4		
			Arthralgia	10 (6.4); 3.5		
			Muscular weakness	10 (6.4); 3.5		
			Back pain	9 (5.8); 3.1		
			Fall	8 (5.1); 2.7		
			Insomnia	8 (5.1); 2.7		
	**>8 weeks (N=470)**	338 (71.9); 86.8	Fatigue	49 (10.4); 5.5	66 (14.0); 7.6	>2 patients<br><br>UTI (n=4; 0.42)<br>MS relapse (n=11; 1.16)<br>Generalized tonic-clonic seizure (n=4; 0.42)
			Headache	48 (10.2); 5.4		
			UTI	45 (9.6); 5.0		
			Depression	35 (7.5); 3.8		
			Lymphopenia	31 (6.6); 3.4		
			Constipation	24 (5.1); 2.6		
Natalizumab exposure	**≤2 years (N=236)**	182 (77.1); 101.0	Fatigue	25 (10.6); 5.6	30 (12.7); 6.8	>1 patient<br><br>UTI (n=2; 0.41)
			Headache	22 (9.3); 4.9		
			UTI	21 (8.9); 4.6		
			Lymphopenia	20 (8.5); 4.3		

			Constipation	16 (6.8); 3.4		MS relapse (n=6; 1.25)<br>Headache (n=2; 0.41)<br>Psychotic disorder (n=2; 0.41)
			Depression	16 (6.8); 3.4		
			Diarrhea	15 (6.4); 3.2		
			Muscular weakness	14 (5.9); 3.0		
			Hypoaesthesia	12 (5.1); 2.5		
			Nausea	12 (5.1); 2.6		
	**>2 years (N=381)**	272 (71.4); 85.4	Fatigue	48 (12.6); 6.9	50 (13.1); 7.1	>2 patients<br><br>UTI (n=4; 0.52)<br>MS relapse (n=7; 0.92)<br>Generalized tonic-clonic seizure (n=3; 0.39)
			Headache	38(10.0); 5.3		
			UTI	36 (9.5); 5.0		
			Depression	25 (6.6); 3.4		
			Lymphopenia	24 (6.3); 3.3		
			Fall	20 (5.3); 2.7		
Exposure to prior immunosuppressive agents	**No (N=574)**	424 (73.9); 93.4	Fatigue	65 (11.3); 6.1	74 (12.9); 7.0	>2 patients<br><br>UTI (n=6; 0.52)<br>MS relapse (n=12; 1.05)<br>Generalized tonic-clonic seizure (n=3; 0.26)<br>Seizure (n=3; 0.26)
			Headache	57 (9.9)%; 5.3		
			UTI	55 (9.6); 5.1		
			Depression	40 (7.0); 3.6		
			Lymphopenia	39 (6.8); 3.5		
			Diarrhea	33 (5.8); 3.0		
	**Yes (N=24)**	12 (50.0); 44.1	Lymphopenia	4 (16.7); 11.0	2 (8.3); 5.1	All SAEs were reported in 1 patient only
			Headache	3 (12.5); 7.3		
			Back pain	2 (8.3); 5.0		
			Constipation	2 (8.3); 4.9		
			Hypoaesthesia	2 (8.3); 4.9		
JCV status	**Negative (N=96)**	76 (79.2) (131.5)	UTI	13 (13.5); 7.5	17 (17.7); 10.4	>1 patient<br><br>UTI (n=2; 1.05)<br>Seizure (n=2; 1.06)
			Headache	10 (10.4); 5.8		
			Lymphopenia	9 (9.4); 5.1		
			Diarrhea	9 (9.4); 5.0		
			Constipation	8 (8.3); 4.5		
			Nasopharyngitis	7 (7.3); 3.9		
			Seizure	7 (7.3); 3.9		
			Depression	6 (6.3); 3.3		
			Hypoaesthesia	6 (6.3); 3.3		
			Muscle spasms	5 (5.2); 2.8		
			Alopecia	5 (5.2); 2.7		
			Arthralgia	5 (5.2); 2.7		
			Nausea	5 (5.2); 2.7		
			Paresthesia	5 (5.2); 2.7		
			URTI	5 (5.2); 2.7		
	**Positive (N=507)**	369 (72.8); 87.7	Fatigue	55 (10.9); 5.8	61 (12.0); 6.4	>2 patients<br><br>UTI (n=3; 0.29)<br>MS relapse (n=12; 1.18)<br>Generalized tonic-clonic seizure (n=4; 0.39)
			Headache	48 (9.5); 5.0		
			UTI	43 (8.5); 4.4		
			Lymphopenia	35 (6.9); 3.6		
			Depression	34 (6.7); 3.4		
			Muscular weakness	28 (5.5); 2.8		

Table S4. Similar publications from the literature.

Author name (year)	Title	No. of patients	Study findings
Cohen et al., 2014	Switching from natalizumab to fingolimod in multiple sclerosis: a French prospective study	333	A survey-based, observational multicenter cohort Study. Results suggest a washout period shorter than 3 months
Comi et al., 2015	**Relapses in patients treated with fingolimod after previous exposure to natalizumab**	254	Fingolimod has the potential to reduce disease reactivation but that timing of treatment initiation may be critical for achieving an optimal effect

De Seze 2013	Reduction of the washout time between natalizumab and fingolimod.	59	Authors recommend reducing as much as possible the delay between the withdrawal of natalizumab and the introduction of fingolimod, to reduce the likelihood of a reactivation of the disease during this high-risk period
Guger et al., 2019	Switching from natalizumab to fingolimod treatment in multiple sclerosis: real life data from the Austrian MS Treatment Registry	195	Switching from natalizumab to fingolimod in a real-world setting is efficacious and safe; results advocate for a short switching gap of 3 months or less
Havla et al., 2013	Fingolimod reduces recurrence of disease activity after natalizumab withdrawal in multiple sclerosis	26	Initiation of fingolimod treatment after natalizumab discontinuation reduces the recurrence of disease activity compared to withdrawal without further immunomodulatory treatment
Jokubaitis et al., 2014	**Fingolimod after natalizumab and the risk of short-term relapse**	89	Authors recommend a maximum 2-month treatment gap for switches to fingolimod to decrease the hazard of relapse
Kappos et al., 2015	**Switching from natalizumab to fingolimod: a randomized, placebo-controlled study in RRMS**	142	Initiating fingolimod therapy 8–12 weeks after natalizumab discontinuation is associated with a lower risk of MRI and clinical disease reactivation than initiation after 16-week washout period
Laroni et al., 2012	Early switch to fingolimod may decrease the risk of disease recurrence after natalizumab interruption.	11	Results suggests that fingolimod might represent a valuable treatment choice for those individuals suspending natalizumab, in order to prevent disease reactivation.
Leurs et al., 2017s	Switching natalizumab to fingolimod within 6 weeks reduces recurrence of disease activity in MS patients	52	Patients with a washout period of >8 weeks had a significant higher recurrence of disease activity compared to patients with a WO period of <6 weeks.
Rinaldi 2012	Switching therapy from natalizumab to fingolimod in relapsing-remitting multiple sclerosis: clinical and magnetic resonance imaging findings	22	Patients were switched to fingolimod after 3 months of washout period. Fingolimod does not exert clinical activity quickly enough to stop MS reactivation after a break from natalizumab.
Vollmer et al., 2018	The impact of very short transition times on switching from Natalizumab to Fingolimod on imaging and clinical effectiveness outcomes in multiple sclerosis	N=117	Results suggest transition periods <1 month are associated with improved clinical outcomes and appears to be safe
Sempere et al., 2013	Switching from natalizumab to fingolimod: an observational study	18	After switching to fingolimod, five of eight patients (63%) experienced clinical relapses, and MRI activity was detected in six of eight patients (75%). No serious side effects were observed with fingolimod.
Fragoso et al., 2016	Safety of switching from natalizumab straight into fingolimod in a group of JCV-positive patients with multiple sclerosis	25	median period of nine months from the medication switch, there were no safety issues to report. The patients had good disease control and no adverse events were reported.
Naegelin et al., 2018	Shortening the washout to 4 weeks when switching from natalizumab to fingolimod and risk of disease reactivation in multiple sclerosis	25	Switching from natalizumab to fingolimod with a washout of 4 weeks – without increasing risk – provides a slightly better protection against recurrent disease activity over 2 years as compared to a washout of 8 weeks
Ziemssen et al., 2019	Long-term real-world evidence for sustained clinical benefits of fingolimod following switch from natalizumab	530	The subgroup analysis from PANGAEA highlights that fingolimod exhibits a favorable benefit–risk profile over 48 months following a switch from natalizumab that is consistent with its benefit–risk profile, following a switch from other DMTs or in patients initiating fingolimod first line. No new safety signals were observed, and the likelihood of relapses between natalizumab discontinuation and fingolimod initiation increased with longer washout durations.
